# ABO Blood Types Are Not Associated with Recurrence After the Surgical Treatment of Chronic Subdural Hematoma—A Retrospective Cohort Study

**DOI:** 10.3390/jcm15062380

**Published:** 2026-03-20

**Authors:** Hussam Hamou, Hani Ridwan, Kimberley Fay-Rodrian, Hans Clusmann, Anke Hoellig, Michael Veldeman

**Affiliations:** 1 Department of Neurosurgery, RWTH Aachen University Hospital, 52074 Aachen, Germany; hhamou@ukaachen.de (H.H.); krodrian@ukaachen.de (K.F.-R.); hclusmann@ukaachen.de (H.C.); ahoellig@ukaachen.de (A.H.); 2 Department of Diagnostic and Interventional Neuroradiology, RWTH Aachen University, 52074 Aachen, Germany; hridwan@ukaachen.de

**Keywords:** chronic subdural hematoma, recurrence, blood types, blood groups, internal architecture, hematoma volume

## Abstract

**Objective**: Chronic subdural hematoma (cSDH) is a common neurosurgical condition with rising incidence in the aging population. Recurrence after surgical evacuation remains frequent, affecting up to one third of patients. Prior studies have proposed an association between ABO blood type and recurrence risk, though the findings are inconsistent. This study aimed to determine whether ABO blood group independently predicts cSDH recurrence after adjusting for clinical and radiological risk factors and to contextualize these findings in the context of previously published studies. **Methods**: We conducted a retrospective cohort study of all consecutive patients surgically treated for cSDH at University Hospital RWTH Aachen between 2015 and 2023. Clinical, laboratory, and imaging data, including hematoma volume, laterality, and architecture, were extracted from medical records. The primary outcome was recurrence requiring reintervention. Associations between ABO blood type and recurrence were assessed using chi-square tests and multivariable logistic regression. A random-effects meta-analysis was performed to integrate our findings with all identified prior studies reporting recurrence stratified by blood type. **Results**: Of 630 treated patients, 488 with documented ABO blood type and recurrence status were included. Recurrence occurred in 31.1% of these patients. ABO distribution matched European population frequencies. Univariate analysis showed no association between blood type and recurrence (*p* = 0.434). Adjusted models likewise showed no significant differences between type A and type B (OR 1.43, 95% CI 0.67–3.00), type AB (OR 2.35, 95% CI 0.74–7.24), and type O (OR 0.95, 95% CI 0.57–1.58). Hematoma architecture remained strongly associated with recurrence (*p* < 0.001). A meta-analysis of available studies similarly demonstrated no association between any ABO blood type and recurrence, with pooled odds ratios near unity across comparisons. **Conclusions**: ABO blood type was not associated with cSDH recurrence in our cohort, and pooled evidence from previously published studies confirms the absence of a meaningful effect. Hematoma architecture and volume remain the most important predictors of recurrence. Based on these results, blood type should not influence postoperative surveillance or counseling, and future work should focus on modifiable biological and imaging-based determinants of recurrence.

## 1. Introduction

Chronic subdural hematoma (cSDH) is a distinct form of intracranial hemorrhage that predominantly affects the elderly. Its incidence has risen steadily over the past decade, currently estimated between 8 and 18 cases per 100,000 individuals annually and reaching up to 36.6 per 100,000 among those aged 80 years or older [1,2,3]. The growing prevalence of cSDH parallels population aging and the increased use of antithrombotic agents, as well as the accumulation of comorbid conditions in older adults [2].

The pathophysiology of cSDH remains incompletely understood. Classically, a minor head injury is thought to produce a small venous bleed from a bridging vein, although the disruption of the dura–arachnoid interface and subsequent formation of a subdural hygroma have also been proposed as initiating events [1,4]. In most cases, these acute subdural collections resolve spontaneously. However, when predisposing factors such as age-related brain atrophy, epilepsy, antithrombotic medication, or alcohol abuse are present, the initial clot may liquefy, giving rise to a slowly enlarging chronic collection. This process reflects a disturbed repair response characterized by local inflammation, fibrinolysis, and angiogenesis. Recent insights highlight injury to dural border cells as a central event that promotes fibroproliferation and the formation of vascularized neomembranes [4]. These membranes consist of an inner layer adjacent to the arachnoid and an outer layer connected to the dura, the latter supplied by meningeal arterial branches. This vascular contribution has led to the development of endovascular middle meningeal artery embolization as a promising therapeutic adjunct [5,6].

Surgical evacuation remains the standard of care in symptomatic patients or when mass effect increases [7]. Because encapsulated or trabeculated hematomas are uncommon, burr hole drainage with irrigation and passive subdural drainage has become the preferred approach. Despite generally favorable outcomes, recurrence requiring reoperation occurs in 5 to 33% of cases and is associated with additional morbidity, prolonged hospitalization, and poorer functional recovery [8,9]. Factors repeatedly linked to recurrence include larger hematoma volume, preoperative antithrombotic therapy, epileptic seizures, persistent postoperative midline shift, and inadequate brain re-expansion [9,10].

More recently, attention has turned to the possible influence of ABO blood type on recurrence risk. Hirai et al. reported a higher recurrence rate in patients with blood group A [11], whereas Hamed et al. found that type O was associated with increased risk [12]. In contrast, in a large Chinese study, involving 1556 patients, the authors observed no significant association [13]. Despite the large sample size, these results may not be extrapolatable to a non-Asian population with a different blood group distribution. These discrepancies may reflect limited sample sizes or the uneven distribution of less common blood types such as B and AB [14].

The present study investigates whether ABO blood group independently predicts cSDH recurrence after adjusting for known clinical and radiological factors. We analyzed a retrospective series of 488 surgically treated patients with documented blood type, paying particular attention to preoperative hematoma architecture as a potential confounder. Given previous conflicting study findings regarding ABO blood type as a risk factor, we further conducted a meta-analysis of all available studies to contextualize our institutional results.

## 2. Methods

### 2.1. Study Design and Patient Population

This retrospective cohort study included all consecutive patients who underwent surgical treatment for chronic subdural hematoma at University Hospital RWTH Aachen between January 2015 and December 2023. The study cohort represents an extension of previously published series from our institution [10,15], with the current analysis focusing specifically on the association between ABO blood type and recurrence risk. Ethical approval was obtained from the local ethics committee (EK20/399 and EK 25/331), and this study was registered with the German Clinical Trials Register (DRKS00025280). Due to the retrospective nature of this investigation, informed consent was waived.

Patients were included if chronic subdural hematoma was confirmed on cranial computed tomography and surgical evacuation was performed via burr hole craniotomy or twist drill craniostomy. Exclusion criteria comprised: the absence of preoperative CT imaging or failure to upload imaging to the institutional archiving system; intracranial hypotension; the contribution of hematoma formation to prior neurosurgical procedures or other cranial trauma; and non-iatrogenic bleeding diatheses including hepatogenic coagulopathy or bleeding disorders.

### 2.2. Data Acquisition

Clinical and radiological data were extracted retrospectively via a systematic review of electronic medical records. Collected variables encompassed patient demographics; a documented history of head trauma; presenting neurological symptoms and deficits; Glasgow Coma Scale score at admission; and pre-existing comorbidities including hypertension, coronary artery disease, cardiac arrhythmias, diabetes mellitus, pulmonary disease, kidney disease, malignancy, prior neurological conditions, alcohol abuse, prior seizure history, peripheral arterial disease, prior deep venous thrombosis or pulmonary embolism, prior myocardial infarction or stroke, and hematologic or hepatic disease. Medication use at the time of admission was documented, specifically the use of antiplatelet agents, dual antiplatelet therapy, oral anticoagulants including vitamin K antagonists and direct oral anticoagulants, angiotensin-converting enzyme inhibitors, angiotensin receptor blockers, and statins. The laboratory values obtained at admission included activated partial thromboplastin time, the international normalized ratio, and platelet count.

The surgical variables documented included the specific technique employed, preoperative coagulation reversal strategies, intraoperative findings, postoperative neurological status, and early complications. ABO blood group, Rhesus factor, and detailed Rhesus phenotype were extracted from hospital transfusion medicine records when available.

Radiological assessment focused on preoperative CT imaging characteristics. Hematoma laterality, maximum width, craniocaudal extent, and volumetric measurements were obtained. Midline shift was measured and recorded. Hematoma architecture was classified using an extended typology system previously developed by our group [16], which categorizes hematomas into eight distinct patterns: bridging, subacute, laminar, trabecular, hyperdense, isodense, hypodense, and sedimented. For analytical purposes, these eight categories were collapsed into four broader classifications: homogeneous (encompassing hypodense, isodense, and hyperdense patterns), organized (including laminar, bridging, and trabecular patterns), sedimented, and subacute.

### 2.3. Outcome Definition

The primary outcome was hematoma recurrence requiring surgical reintervention. Patients were routinely discharged following complete symptom resolution or when postoperative imaging demonstrated the resolution of mass effect. Surveillance imaging was performed at 14- to 21-day intervals after initial treatment and continued biweekly until radiological remission was confirmed. Recurrence was defined as the volumetric enlargement of residual or newly accumulated hematoma with radiological mass effect or new onset or worsening of neurological symptoms necessitating repeat surgical evacuation. Reintervention consisted of repeat burr hole craniotomy with the placement of subdural silicone drains.

### 2.4. Meta-Analysis

To synthesize evidence across all available studies examining ABO blood type and chronic subdural hematoma recurrence, we conducted a systematic meta-analysis. Given the anticipated small number of eligible studies on this specific topic, implementing a full PRISMA workflow was not considered practical. A targeted literature search was performed to capture all available studies reporting recurrence stratified by ABO blood type, which were then included in the meta-analysis. A systematic search of PubMed, Embase, and Web of Science was conducted using the terms ‘chronic subdural hematoma’, ‘recurrence’, ‘blood type’, and ‘ABO’. Searches covered all publications from database inception to January 2025. Two reviewers (HH and MV) independently screened titles, abstracts, and full texts. Inclusion criteria: (1) clinical studies reporting the recurrence rates of cSDH stratified by ABO blood type; (2) adult patients; (3) extractable recurrence data. Exclusion criteria: reviews, case reports, or studies lacking stratified recurrence data. Disagreements were resolved by consensus. We identified three prior studies: Hirai et al. (2021, n = 350) [11], Hamed et al. (2023, n = 229) [12], and Ou et al. (2022, n = 1556) [13]. Raw recurrence data stratified by blood type were extracted from each study. To enable the pooling of effect estimates, all studies were standardized to use blood type A as the common reference category. Odds ratios and standard errors were calculated for each blood type comparison (B vs. A, AB vs. A, O vs. A) within each study using standard two-by-two contingency table methods. Random-effects meta-analysis using the DerSimonian–Laird method was performed separately for each blood type comparison to account for potential between-study heterogeneity. Heterogeneity was quantified using the I^2^ statistic and Cochran’s Q test. Forest plots were generated to visualize individual study estimates alongside pooled effect estimates with 95% confidence intervals.

### 2.5. Statistical Analysis

All analyses were performed using R version 4.4.3 (R Foundation for Statistical Computing, Vienna, Austria) within RStudio Version 2025.09.1+401 (Posit Software, PBC, Boston, MA, USA). The primary packages utilized included readxl (Version 1.4.5), tidyverse (Version 2.0.0), broom (Version 1.0.12), binom (Version 1-1-1.1), car (Version 3.1-5), pROC (Version 1.19.0.1), and ResourceSelection (Version 0.3-6). Meta-analytic procedures were performed using the metafor (Version 4.8-0) and meta (Version 8.2-1) packages. Patients with missing recurrence status were excluded from analysis as this represented the primary outcome.

Missingness patterns were systematically evaluated for key variables. Chi-square tests and *t*-tests assessed whether missing data were associated with recurrence or patient characteristics. ABO blood type missingness showed no association with recurrence (missing completely at random), supporting complete case analysis. In contrast, Rhesus factor missingness was strongly associated with recurrence status (missing not at random), as complete Rhesus typing was more frequently performed in patients undergoing reoperation due to blood product preparation. Consequently, the Rhesus factor was excluded from primary analyses. Complete case analysis was employed for multivariable models given adequate sample retention and minimal confounding variable missingness.

Continuous variables were summarized using means and standard deviations (SDs) or medians and interquartile ranges (IQRs, Q1–Q2) based on data distribution. Categorical variables were presented as frequencies and percentages. The ABO blood type distribution in our cohort proved compared to expected European population frequencies [17].

The association between ABO blood type and recurrence was evaluated using Pearson’s chi-square test. Recurrence rates for each blood type were calculated with 95% confidence intervals using the Wilson score method. A pairwise comparison of types A and O employed two-proportion z-tests. Associations between extended hematoma type, other clinical variables, and recurrence were similarly assessed using chi-square tests for categorical variables and *t*-tests or Mann–Whitney U tests for continuous variables as appropriate.

Logistic regression models assessed the independent association between ABO blood type and recurrence while controlling for confounders. Blood type A served as the reference category. An iterative model-building approach was employed, beginning with a base model including blood type, demographics (age, sex), and anticoagulation status (antiplatelet therapy, oral anticoagulation). Subsequent models progressively incorporated imaging characteristics (preoperative volume, laterality, extended hematoma type), laboratory values (international normalized ratio, platelet count), and comorbidities (hypertension, diabetes, alcohol abuse, trauma history).

Likelihood ratio tests (LRTs) compared nested models to determine whether additional variable groups significantly improved model fit, guiding the selection of the most parsimonious final model. Variance inflation factors (VIFs) assessed multicollinearity, with values above 5 indicating concern. Model discrimination was quantified using the area under the receiver operating characteristic curve. The Hosmer–Lemeshow test evaluated model calibration.

The results were reported as odds ratios (ORs) with 95% confidence intervals (CIs). Given three blood type comparisons (B, AB, and O versus A), Bonferroni correction established a significance threshold of 0.017. All tests were two-sided with a conventional alpha of 0.05 unless otherwise specified.

## 3. Results

### 3.1. Patient Selection and Cohort Characteristics

**Figure 1:**
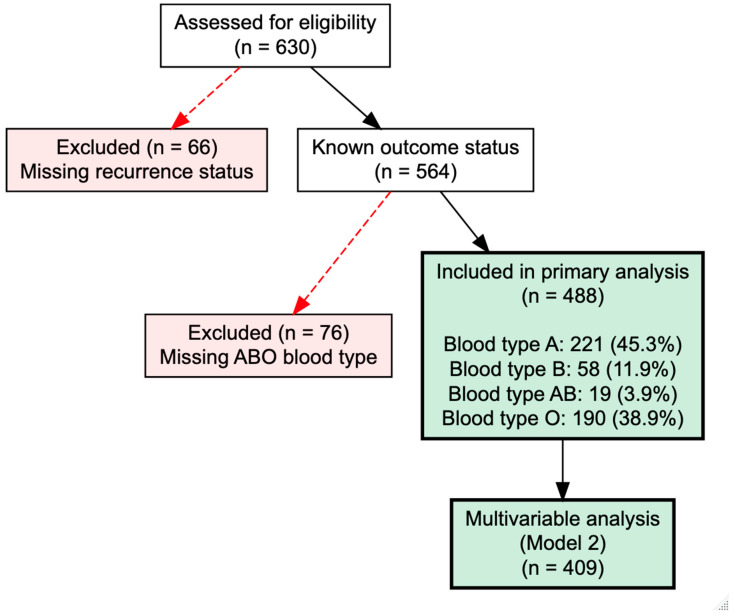
Inclusion flow chart. Diagram illustrating patient selection for inclusion in final analyses.

Between January 2015 and December 2023, 630 consecutive patients underwent surgical treatment for chronic subdural hematoma at our institution. Of these, 66 patients were excluded due to missing recurrence status, resulting in 564 patients with documented outcome data. Among this cohort, ABO blood type information was unavailable for 76 patients, yielding 488 patients with complete blood type and recurrence data for primary analysis. The ABO blood type distribution comprised 221 patients with type A (45.3%), 190 with type O (38.9%), 58 with type B (11.9%), and 19 with type AB (3.9%). This distribution closely approximated expected European population frequencies. For multivariable analysis adjusting for demographics and anticoagulation status, 486 patients had complete data. After incorporating imaging variables including extended hematoma type classification, 409 patients remained for adjusted analysis. The final multivariable model including laboratory values and comorbidities comprised 402 patients. The overall recurrence rate requiring surgical reintervention was 30.1% (170 of 564 patients with known outcome). Among patients with documented ABO blood type, the recurrence rate was 31.1% (152 of 488 patients). The patient inclusion process is illustrated as a flow chart in Figure 1.

### 3.2. Univariate Analysis

Univariate analysis revealed no significant association between ABO blood type and recurrence (χ^2^ = 2.735; *p* = 0.434). Recurrence rates by blood group were as follows: type A, 29.4% (65 of 221 patients; 95% CI, 23.8–35.7%); type B, 37.9% (22 of 58 patients; 95% CI, 26.6–50.8%); type AB, 42.1% (8 of 19 patients; 95% CI, 23.1–63.7%); and type O, 30.0% (57 of 190 patients; 95% CI, 23.9–36.9%). A direct comparison of types A and O, the two most prevalent groups implicated in the prior literature, demonstrated nearly identical recurrence rates (risk ratio = 0.98; *p* = 0.983). While type AB showed a numerically higher recurrence rate, the confidence interval was exceptionally wide due to the small sample size (n = 19), and this difference did not reach statistical significance. Conversely, hematoma architecture on preoperative CT imaging showed a strong association with recurrence (*p* < 0.001, Table 1). Organized hematomas demonstrated the lowest recurrence rate (19.4%), followed by subacute hematomas (16.4%), while homogeneous and sedimented hematomas exhibited substantially higher recurrence rates of 43.4% and 53.3%, respectively. Larger preoperative hematoma volume was also associated with increased recurrence risk (*p* = 0.079).

### 3.3. Multivariable Analysis

Multivariable logistic regression analysis (Model 2) adjusting for demographics, anticoagulation status, hematoma volume, laterality, and architecture included 409 patients (Table 2). After controlling for these confounders, no significant association was observed between ABO blood type and recurrence. Compared to blood type A as the reference, the adjusted odds ratios were 1.43 (95% CI, 0.67–3.00; *p* = 0.345) for type B, 2.35 (95% CI, 0.74–7.24; *p* = 0.138) for type AB, and 0.95 (95% CI, 0.57–1.58; *p* = 0.844) for type O. Although type AB demonstrated an elevated point estimate, this finding was based on only 16 patients and should be interpreted with caution. The application of Bonferroni correction for multiple comparisons (adjusted α = 0.017) confirmed no significant associations. Hematoma architecture remained the strongest predictor of recurrence in the adjusted model. Organized hematomas showed markedly reduced recurrence risk (OR = 0.28; 95% CI, 0.16–0.48; *p* < 0.001) compared to homogeneous hematomas, as did subacute hematomas (OR = 0.24; 95% CI, 0.10–0.50; *p* < 0.001). Larger preoperative hematoma volume independently predicted increased recurrence (OR = 1.01 per mL; 95% CI, 1.00–1.01; *p* = 0.004). The model demonstrated acceptable discrimination (area under the curve = 0.724) and good calibration (Hosmer–Lemeshow *p* = 0.435). LRTs confirmed that the addition of imaging variables and hematoma architecture significantly improved model fit compared to demographic variables alone (*p* < 0.001), while the subsequent addition of laboratory values or comorbidities did not provide significant incremental benefit (*p* = 0.931 and *p* = 0.515, respectively). VIFs for all covariates remained below 5, indicating no concerning multicollinearity.

### 3.4. Meta-Analysis

**Figure 2:**
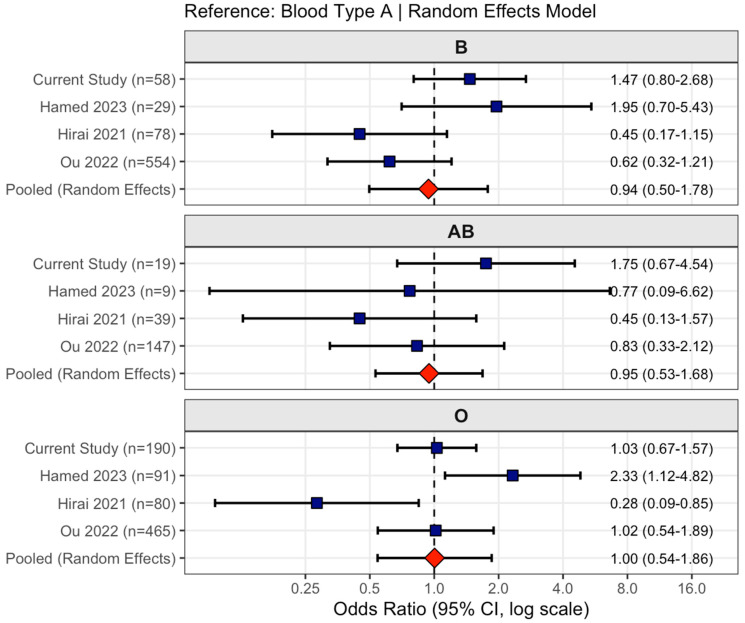
Meta-analysis of ABO blood type and chronic subdural hematoma recurrence across four studies [11,12,13]. Forest plots showing odds ratios (ORs) and 95% confidence intervals (CIs) for recurrence risk by blood type compared to type A as reference. Individual study estimates (blue squares) and pooled random-effects estimates (red diamonds) are displayed separately for blood types B, AB, and O. Square size represents sample size of blood type group within each study. Vertical dashed line at OR = 1.0 indicates no association. Pooled estimates demonstrate no significant associations: type B OR = 0.94 (95% CI: 0.50–1.78), type AB OR = 0.95 (95% CI: 0.53–1.68), and type O OR = 1.00 (95% CI: 0.54–1.86). Substantial heterogeneity was observed for types B (I^2^ = 62%) and O (I^2^ = 70%), while type AB showed minimal heterogeneity (I^2^ = 1.6%). Combined analysis included 2623 patients with 301 recurrence events.

The meta-analysis incorporating all four studies (combined n = 2623 patients, 301 recurrence events) demonstrated no association between ABO blood type and cSDH recurrence. This analysis is depicted as a forest plot in Figure 2. Using random-effects models with blood type A as the reference, the pooled odds ratios were 0.94 (95% CI: 0.50–1.78, *p* = 0.850) for type B, 0.95 (95% CI: 0.53–1.68, *p* = 0.849) for type AB, and 1.00 (95% CI: 0.54–1.86, *p* = 0.989) for type O. All pooled estimates had confidence intervals crossing unity, indicating no significant associations. Substantial between-study heterogeneity was observed for type B (I^2^ = 62%, Q *p* = 0.047) and type O (I^2^ = 70%, Q *p* = 0.018), reflecting contradictory findings in smaller studies. In contrast, type AB showed minimal heterogeneity (I^2^ = 1.6%, Q *p* = 0.384), with all studies consistently demonstrating null associations. Notably, for type O, the two largest studies comprising over two thousand patients (Ou et al. [13] and the current study) both yielded odds ratios approximating 1.0, while smaller studies reported conflicting estimates ranging from 0.28 to 2.33. The pooled estimates were very close to unity for type O (OR = 1.00) despite individual study variation, underscoring that larger, adequately powered investigations consistently fail to detect associations between ABO blood type and recurrence risk.

## 4. Discussion

This retrospective cohort study evaluated the association between ABO blood type and recurrence risk following the surgical treatment of chronic subdural hematoma. Our primary objective was to determine whether blood type serves as an independent predictor of recurrence after adjusting for established clinical and radiological risk factors. In a cohort of over four hundred patients with documented ABO blood type and recurrence status, we found no significant association between blood group and the likelihood of requiring surgical reintervention. This null finding remained consistent across both univariate and multivariable analyses. Specifically, a direct comparison of blood types A and O, the two groups implicated in the prior conflicting literature, revealed virtually identical recurrence rates. While type AB demonstrated a numerically higher recurrence rate, this observation was based on a very small sample and likely represents chance variation rather than a true biological effect. In contrast to blood type, hematoma architecture on preoperative CT imaging emerged as the strongest predictor of recurrence, with organized and subacute hematomas showing substantially lower recurrence risk compared to homogeneous or sedimented patterns. These findings suggest that ABO blood type does not play a clinically meaningful role in cSDH recurrence and that previously reported associations in smaller cohorts likely reflect statistical artifacts arising from inadequate sample sizes, particularly for rarer blood types.

The biological mechanisms by which ABO blood group might theoretically influence cSDH recurrence warrant brief consideration. ABO antigens are known to modulate the plasma levels of von Willebrand factor and factor VIII, with non-O blood types associated with higher prothrombotic activity and increased venous thromboembolism risk [18,19]. These coagulation differences are well-documented across clinical settings and are partially mediated through ADAMTS13-dependent clearance mechanisms [18]. In the context of cSDH, one might hypothesize that blood type-related differences in coagulation or inflammatory signaling could influence neomembrane formation, fibrinolysis, or hematoma re-accumulation. However, the pathophysiology of cSDH recurrence is also governed by local factors, such as hematoma architecture, volume, and the degree of brain re-expansion. This may explain why blood type differences in thromboembolic disease, which are detectable at the population level, do not necessarily translate into meaningful differences in cSDH recurrence risk.

The relationship between ABO blood type and cSDH recurrence has been investigated in three prior studies with conflicting results. Hirai and colleagues reported that blood type A was associated with increased recurrence risk in a cohort of over three hundred patients, identifying type A as an independent predictor with an OR approaching three in multivariable analysis [11]. Their study design included careful follow-up protocols and adjustment for relevant confounders including thrombocytopenia and coagulopathy. In contrast, Hamed et al. identified blood type O as a risk factor for recurrence in a cohort of over two hundred patients, reporting an approximately twofold increased odds of reoperation within twelve weeks [12]. Their analysis also controlled for anticoagulation status and patient comorbidities. Most recently, Ou and colleagues examined nearly sixteen hundred patients and found no association between any ABO blood type and recurrence risk, concluding that blood type should not be considered in clinical decision-making regarding cSDH management [13]. This latter finding aligns with our current results. To contextualize these divergent findings, we performed a meta-analysis incorporating all available studies together with our own data. Across more than 2600 patients, pooled random-effects models demonstrated no significant association between any ABO blood type and recurrence risk.

Taken together, several methodological considerations may explain the discrepant findings between studies. Both studies reporting positive associations had relatively modest sample sizes with limited numbers of recurrence events, which is particularly problematic when analyzing categorical variables with multiple levels. In the study identifying type A as a risk factor, only thirty-seven total recurrence events occurred across all blood types [11]. Similarly, in the cohort identifying type O as a predictor, blood type AB comprised only nine patients with a single recurrence event, yielding a confidence interval spanning from near zero to nearly fifty percent [12]. Type B included fewer than thirty patients, also resulting in substantial statistical imprecision. With such small numbers in individual blood type categories, random variation can easily produce apparent associations that do not reflect true biological relationships. Furthermore, neither positive study adjusted for hematoma architecture on imaging, which our analysis and prior work have demonstrated to be among the strongest predictors of recurrence [16].

Our study addresses several of these limitations. With nearly five hundred patients with documented blood type, our cohort exceeds the sample sizes of both studies reporting positive findings. Importantly, our analysis explicitly adjusted for hematoma architecture using a validated classification system, along with other established risk factors including hematoma volume, anticoagulation status, and patient demographics. We also applied appropriate corrections for multiple comparisons, setting our significance threshold to account for testing three blood type comparisons simultaneously. The consistency of our null findings with the largest prior investigation, combined with the methodological limitations of the smaller positive studies, suggests that previously reported associations may represent statistical artifacts rather than true biological phenomena. The biological plausibility of blood type affecting cSDH recurrence also remains unclear, as ABO antigens’ potential mechanisms influencing hemorrhage evolution, inflammatory response, or membrane formation have not been convincingly demonstrated in this clinical context.

## 5. Limitations

This study has several important limitations. The retrospective design introduces potential for unmeasured confounding, and variables such as postoperative mobilization protocols and the precise timing of anticoagulation resumption were not systematically captured. Although nearly five hundred patients had documented ABO blood type, missingness reduced the size of our analytical cohort. However, blood type missingness was not associated with recurrence risk, suggesting minimal selection bias. Conversely, Rhesus factor data showed informative missingness, as complete typing occurred more frequently in patients undergoing reoperation, precluding meaningful Rhesus analysis.

Only nineteen patients had blood type AB, resulting in wide CIs and substantial statistical uncertainty for this subgroup. Post hoc power analysis confirmed that the type AB subgroup was substantially underpowered for the AB versus A comparison (power 10.1% at α = 0.017), and the results for this subgroup should therefore be interpreted with caution; sensitivity analysis excluding type AB patients confirmed that the findings for the remaining blood type comparisons were, however, unaffected. While the application of multiple comparison corrections addresses this concern from an inferential perspective, we cannot definitively exclude associations in this rare blood type. Our single-center cohort may limit generalizability to populations with different ethnic compositions and blood type distributions. Finally, our hematoma architecture classification, while validated in prior work from our institution, has not been universally adopted and may not be directly comparable to systems used elsewhere.

The meta-analysis was constrained by the small number of eligible studies, which limits statistical power and increases vulnerability to small-study effects. Heterogeneity was notable in some comparisons, likely reflecting methodological differences and the absence of imaging-based hematoma characterization in prior cohorts. Because fewer than ten studies were available, publication bias could not be assessed using funnel plots, consistent with Cochrane guidance. These factors reduce the precision of pooled estimates, though the largest studies consistently showed no association between ABO blood type and recurrence.

## 6. Conclusions

This study found no association between ABO blood type and recurrence risk following the surgical treatment of chronic subdural hematoma. After adjusting for established clinical and radiological predictors, including hematoma architecture, volume, and anticoagulation status, blood type did not independently predict the likelihood of requiring reoperation. Our findings align with the largest prior investigation and contrast with smaller studies reporting associations with type A or O, which likely reflect statistical artifacts arising from limited sample sizes and inadequate adjustment for confounding variables. Hematoma architecture on preoperative imaging emerged as the strongest predictor of recurrence, with organized and subacute patterns demonstrating substantially reduced risk compared to homogeneous or sedimented hematomas. These results suggest that ABO blood type should not influence clinical decision-making regarding surveillance intensity or recurrence risk counseling in patients undergoing the surgical evacuation of chronic subdural hematoma. Future research should focus on further elucidating the biological mechanisms underlying hematoma organization and identifying modifiable risk factors that may reduce recurrence rates in this increasingly prevalent condition.

## Figures and Tables

**Table 1:** Baseline characteristics stratified by ABO blood group. Data are presented as mean (standard deviation) for continuous variables and n (%) for categorical variables. *p*-values were calculated using analysis of variance for continuous variables and chi-square tests for categorical variables.

	ABO Blood Group					
Characteristic	A	B	AB	O	Overall	*p*-Value ^2^
	N = 221 ^1^	N = 58 ^1^	N = 19 ^1^	N = 190 ^1^	N = 488 ^1^	
**Age, years**	76.0 (11.7)	74.1 (13.0)	75.7 (15.9)	74.0 (12.1)	75.0 (12.2)	0.241
**Sex**						0.389
Female	78 (35.3%)	19 (32.8%)	3 (15.8%)	65 (34.2%)	165 (33.8%)	
**Glasgow Coma Scale**	14.4 (1.6)	14.0 (2.2)	14.1 (1.6)	14.3 (1.9)	14.3 (1.8)	0.467
**Documented head trauma**	164 (74.2%)	40 (69.0%)	11 (57.9%)	137 (72.1%)	352 (72.1%)	0.444
**Antiplatelet therapy**	81 (36.7%)	14 (24.1%)	7 (36.8%)	60 (31.6%)	162 (33.2%)	0.329
**Oral anticoagulation**	33 (14.9%)	11 (19.0%)	2 (10.5%)	29 (15.3%)	75 (15.4%)	0.79
**Statin treatment**	70 (31.7%)	17 (29.3%)	9 (47.4%)	62 (32.6%)	158 (32.4%)	0.520
**Hypertension**	148 (67.0%)	32 (55.2%)	11 (57.9%)	114 (60.0%)	305 (62.6%)	0.246
**Diabetes mellitus**	34 (15.4%)	9 (15.5%)	6 (31.6%)	36 (18.9%)	85 (17.4%)	0.297
**Alcohol abuse**	10 (4.5%)	3 (5.2%)	0 (0.0%)	6 (3.2%)	19 (3.9%)	0.669
**Preoperative volume, mL**	143.2 (70.2)	148.4 (66.4)	141.2 (52.9)	138.5 (66.2)	141.9 (67.5)	0.579
Missing	9	4	0	10	23	
**Maximum volume, mL**	137.2 (65.2)	147.5 (51.4)	142.5 (52.8)	128.9 (56.7)	135.2 (60.1)	0.1
Missing	36	14	3	22	75	
**Bilateral hematoma**	48 (21.7%)	17 (29.3%)	3 (15.8%)	61 (32.1%)	129 (26.4%)	0.071
**Hematoma architecture**						0.354
Homogeneous	84 (38.5%)	29 (50.9%)	7 (36.8%)	85 (45.2%)	205 (42.5%)	
Organized	87 (39.9%)	14 (24.6%)	7 (36.8%)	72 (38.3%)	180 (37.3%)	
Sedimented	12 (5.5%)	6 (10.5%)	1 (5.3%)	11 (5.9%)	30 (6.2%)	
Subacute	35 (16.1%)	8 (14.0%)	4 (21.1%)	20 (10.6%)	67 (13.9%)	
Missing	3	1	0	2	6	
**Midline shift present**	161 (72.9%)	39 (67.2%)	13 (68.4%)	127 (66.8%)	340 (69.7.3%)	0.467
**International normalized ratio**	1.1 (0.6)	1.2 (0.4)	1.1 (0.1)	1.2 (0.6)	1.2 (0.6)	0.861
Missing	4	0	0	5	9	
**Platelet count, ×10^9^/L**	246.4 (92.2)	246.8 (80.5)	271.9 (59.4)	232.1 (84.9)	241.8 (87.3)	0.045
Missing	4	0	1	3	8	
**Recurrence requiring reoperation**	65 (29.4%)	22 (37.9%)	8 (42.1%)	57 (30.0%)	152 (31.1%)	0.434

^1^ Mean (SD) or n (%), ^2^ Kruskal–Wallis rank sum test; Pearson’s chi-squared test.

**Table 2:** Multivariable logistic regression analysis of recurrence risk. Odds ratios (ORs) and 95% confidence intervals (CIs) from multivariable logistic regression analysis adjusting for demographics, anticoagulation status, hematoma volume, laterality, and architecture (n = 409). Reference categories: blood type A, male sex, unilateral hematoma, homogeneous architecture.

Variable	OR	95% CI	*p*-Value
**ABO blood group**			0.35
A			
B	1.4	0.67–3.00	
AB	2.4	0.74–7.24	
O	1	0.57–1.58	
**Age (per year)**	1	0.99–1.03	0.341
**Sex**			0.188
female	1.4	0.85–2.29	
**Antiplatelet therapy**			0.416
yes	0.8	0.48–1.34	
**Oral anticoagulation**			0.084
yes	0.6	0.28–1.08	
**Hematoma architecture**			<0.001
homogeneous			
organized	0.3	0.16–0.48	
sedimented	1.7	0.69–4.07	
subacute	0.2	0.10–0.50	
**Preoperative volume (per mL)**	1	1.00–1.01	0.004
**Hematoma laterality**			0.205
bilateral	0.7	0.39–1.22	

Abbreviations: CI = Confidence Interval; OR = Odds Ratio. Reference categories: blood type A, male sex, unilateral hematoma, homogeneous architecture.

## Data Availability

The raw data of this analysis can be made available by the authors to any qualified researcher upon reasonable request.

## Abbreviations

The following abbreviations are used in this manuscript:

ABO	Blood group system based on the presence of A and B antigens
AUC	Area under the receiver operating characteristic curve
CI	Confidence interval
cSDH	Chronic subdural hematoma
CT	Computed tomography
DRKS	Deutsches Register Klinischer Studien (German Clinical Trials Register)
OR	Odds ratio
SD	Standard deviation
VIF	Variance inflation factor
